# Is the genomic translational pipeline being disrupted?

**DOI:** 10.1186/s40246-015-0032-4

**Published:** 2015-06-14

**Authors:** Marc S. Williams

**Affiliations:** Genomic Medicine Institute, Geisinger Health System, 100 N Academy Dr., Danville, PA 17822-2620 USA

**Keywords:** Genomic medicine, Disruption, Innovation, Translational pipeline, Direct-to-consumer, Patient powered research

## Abstract

The translational pipeline for genomic medicine has been well defined. However, as with any rapidly changing technology, innovations are difficult to predict leading to the potential to disrupt anticipated translation. Examples of potential disruptors such as laboratory-developed tests, direct-to-consumer testing, and patient-centered research are presented. Awareness of the disruptive nature of innovative approaches is necessary if these innovations are to be incorporated into current practice.

In 2011, the National Human Genome Research Institute (NHGRI) released its new strategic plan [1]. This plan included an emphasis on the implementation of genomic medicine in the clinic. The strategic plan has guided the NHGRI’s funding of several projects such as the electronic Medical Records and Genomics (eMERGE) and Implementing Genomics in Practice (IGNITE) networks that include implementation as part of the project goals [2]. Implementation represents the end of a translational pipeline that starts with discovery research, proceeds through candidate health applications and translation into practice recommendations and guidelines to full implementation that results in measurable improvement in health outcomes [3]. The current organization of research and attendant resources assumes that the translation pipeline is the only way for genomic medicine to move into practice. This discounts the possibility that outside factors may impact and potentially disrupt this pipeline. This commentary will present current examples and explore their potential for disruption.

## What is disruption?

Disruption, according to the dictionary, means something “radically different” or “upsetting.” However, disruption as used in business refers to an innovation that makes things simpler and more affordable resulting in a transformation of the industry. The example frequently used is computers. In the 1970s, computers were large, mainframe units that were expensive and only available to large businesses or government entities. A handful of companies like International Business Machines (IBM) and Control Data controlled the industry and were very successful. When the first personal computers were introduced, they were significantly less powerful than the mainframes and were of little interest to either the existing computer companies or their clients. However, putting low-cost computers into the hands of large numbers of people democratized the industry and allowed rapid innovation that in 10 years put all of the large computer companies out of business. The exception was IBM which created an entirely separate business unit devoted to personal computers in recognition that existing entities are unlikely to be able to adapt to the disruptive technology.

## Disruption in genomics

### Genetic testing in the clinic

One the first disruptions to the translation pipeline involved clinical genetic tests. In contrast to medications that must go through a formal approval process, in the United States (US), laboratory tests can come to the market with little regulatory oversight. Rather than following the translational process to develop evidence of improved health outcomes prior to introduction to the market, tests come to market based on early evidence of clinical validity or disease/gene associations. In some cases, little evidence exists regarding analytic test performance. The vast majority of clinical genetic tests have come to market through this way. Much has been written about the lack of incentives and reimbursement for test developers to generate evidence of utility [4] which has led to consideration of a regulatory framework to drive tests back through the pipeline so that evidence of utility can be confirmed. This is fraught with challenges as well as concerns about how adaptable a regulatory framework will be in a field where the technical advances take place at such a rapid pace [5].

### Direct-to-consumer genetics

Almost 10 years ago, several direct-to-consumer genetic testing companies appeared. These offerings exhibited many of the hallmarks of a disruptive innovation. They were provided outside the usual medical infrastructure at a cost that was significantly less expensive than clinically available tests. The tests themselves offered less utility with much of the information provided not relevant to health. Bypassing the physician caused a considerable amount of consternation in both the medical and ethics communities. All but one of the companies disappeared from the market relatively quickly. Reasons for this included the lack of perceived utility, a price point that exceeded the consumer’s willingness to pay and inability to realize a sustainable business model—factors that appear to have been more significant than the ethical concerns. The remaining company, 23andMe has managed to remain in the market despite running afoul of the US Food and Drug Administration. Outside of a larger capital commitment, 23andMe has pioneered several innovations that have the potential to alter the translational pipeline. 23andMe participants are offered the opportunity to complete a variety of surveys that have led to an increasingly rich resource of participant entered data. This is being used to power novel research methodologies. In partnership with other organizations around Parkinson’s disease, the database was used to discover six new susceptibility loci for Parkinson’s disease [6]. The company is currently participating in 230 different research studies. If this model proves successful through the development of innovative methods that allow more research to be done at a lower cost, this could significantly alter the approach of traditional research funders.

### Patients take charge

To this point, the scientific research agenda has been determined by the investigators and funding agencies. While this has resulted in important progress, the lack of involvement of patients as anything other than participants in the research has arguably impacted the implementation of research results given that some researcher-defined outcomes are not considered relevant by patients. In addition, many disorders have had little research done as they have not been prioritized by the scientists and funders. The advent of social media and the internet has allowed patients to begin to aggregate. Initially, the intent of the groups was to connect patients and families to share information and provide support. However, it was soon recognized that these groups provided an opportunity to research these conditions. This has been particularly fruitful in ultra-rare genetic diseases. However, this model still depended on identifying academic researchers and traditional funding sources which were not always available.

The first large scale effort that leveraged the internet and social media was PatientsLikeMe™. Founded in 2004, PatientsLikeMe™ is a patient-powered research network. Originally focused on amyotrophic lateral sclerosis, (ALS), PatientsLikeMe™ has expanded to over 2000 different disorders. Using an online platform, PatientsLikeMe™ collects longitudinal patient-supplied data across a wide range of parameters including symptom severity, triggers, and response to therapies. An online data sharing platform allows rapid dissemination of the submitted information, and a search engine provides information about clinical trials throughout the world. PatientsLikeMe™ maintains an internal staff of researchers. A recent demonstration of the disruptive potential of this resource involved ALS and lithium. The results of a small study suggested that lithium might slow the progression of the disease. Many ALS patients began using lithium off-label, and a subset of these regularly entered data in the PatientsLikeMe™. In just 9 months, using the self-reported data of nearly 350 ALS patients PatientsLikeMe™ demonstrated that lithium did not slow the progress of the disease [7]. While much remains to be learned about the role and robustness of this type of research, it is clear that there is no other research entity at present that could have conducted this study at the cost and within the timeframe in which it was performed. A recent editorial examined the potential impact of this as a disruptor of research [8]. Of more concern to traditional research are reports that ALS participants in double-blinded prospective randomized controlled trials shared information on their clinical response to these sites effectively unblinding the arms [9]. As one affected patient stated, “We simply don’t have time to wait for the results of ‘clinical trials.’ Our life spans are much shorter than the ‘Food and Drug Administration’ approval process.” The impact on the research findings is yet to be studied [10].

In some ways, the impact of these patient-centered efforts has already achieved a significant impact on research funding. The Affordable Care Act created a new funding organization, the Patient-Centered Outcomes Research Institute (PCORI) which has as one of its guiding principles, a commitment to involving patients both as scientific partners and as part of the review process. Recently, a large infrastructure funding opportunity from PCORI called PCORnet is funding up to 22 Patient-Powered Research Networks (PPRNs). These PPRNs “…comprise patients and/or caregivers who are motivated to build an ideal network and play an active role in patient-centered comparative effectiveness research (CER)” [11]. PCORI is facilitating the interaction between these PPRNs, and traditional investigator lead Clinical Data Research Networks to create a new model for network-based health care research.

This model is emerging in genomic medicine. GenomeConnect is a patient-powered registry that is using patient- and family-entered data to gather rich information on a variety of genetic conditions. This uses a fully consented model for the research using the data, which is de-identified for the protection of the data contributors. Genetic test report results are uploaded and associated with the clinical data to facilitate research into the phenotypes associated with specific genetic variants. This project is now receiving funding through the National Institutes of Health’s (NIH) Clinical Genome Resource project, one of the first examples of a traditional research funder studying the role and impact of patient- and family-entered data to enhance a publicly available genomic resource. While still early, one project, Simons VIP which is studying the genetic causes of autism enrolled over 400 patients and families in 2014. Four out of five of the registrants indicated an interest in participating in research and therapeutic trials. Genetics is also represented in the PCORI PPRNs as three of the funded networks are based around a genetic disease with one of the three using GenomeConnect for data contribution [12].

Support for this as an emerging model came from the recent NIH Precision Medicine stakeholder conference where one the strongest recommendations to come out of the meeting was the need to involve patients not only as participants but as partners in designing and executing the project [13].

## Conclusion

There are compelling reasons why a robust translational pipeline for genomic medicine is desirable. However, in a field of rapidly changing technology and knowledge, innovation may result in translational pathways that circumvent and disrupt the assumed pipeline. While this may not be entirely predictable or preventable, awareness of this potential may allow for incorporation of some of these innovations in current programs as is seen in some of the examples provided. Ultimately, this has the potential to increase the speed and value of the translation of genomic medicine into practice.
